# Real Spinal Cord Injury Without Radiographic Abnormality (SCIWORA) in Pediatrics: A Clinical Case Report and Literature Review

**DOI:** 10.7759/cureus.50491

**Published:** 2023-12-13

**Authors:** Joao Meira Goncalves, Sara Carvalho, Ana Isabel Silva, Josué Pereira, Patricia Polónia

**Affiliations:** 1 Neurosurgery, Centro Hospitalar Universitário de São João, Porto, PRT; 2 Neuroradiology, Centro Hospitalar Universitário de São João, Porto, PRT; 3 Physical Medicine and Rehabilitation, Centro Hospitalar Universitário de São João, Porto, PRT

**Keywords:** pediatric trauma, pediatric emergency, pediatric spine, spinal cord injury, sciwora

## Abstract

Spinal cord injury without radiological abnormality (SCIWORA) was first reported in 1974. The term was used to define “clinical symptoms of traumatic myelopathy without signs of fracture or spine instability on X-ray or CT scan.” With the emergence of MRI, the gold standard method to identify spinal cord injuries, about two-thirds of former SCIWORA cases were found to have pathological findings, and, as such, the term has taken on an ambiguous meaning in the literature.

We describe the clinical case of a 17-year-old boy who was admitted to the emergency department of a tertiary hospital after a fall during a soccer game. He suffered spinal and cranioencephalic trauma. A few minutes later, the boy began to show decreased strength in the right upper limb and lower limbs, as well as changes in sensation in the right hemibody.

On objective examination, the boy presented a Glasgow Coma Scale score of 15 and the American Spinal Injury Association Impairment Scale D, with partial improvement of initial symptoms of monoparesis of the right lower limb. There were no other changes, specifically at the sensory level. The patient underwent a CT and MRI of the spine that showed no fractures, instability, or appreciable medullary signal changes. Electromyography was normal. Based on the clinical history and imaging findings, real SCIWORA was diagnosed. The patient was admitted to an inpatient rehabilitation program. At a follow-up visit two months later, a complete reversal of signs and symptoms was confirmed.

The prognosis of this pathology depends on the extent of the spinal cord injury, as evidenced by MRI. Although neurological improvement when severe deficit is present at initial presentation is unlikely, most patients with incomplete neurological damage show good recovery. The absence of visible changes on MRI is associated with a better prognosis.

## Introduction

Spinal cord injury without radiological abnormality (SCIWORA) is often described as the manifestation of acute traumatic myelopathy despite normal X-rays and CT scans [1]. It predominantly affects the cervical spine [2-4]. The incidence of SCIWORA was found to be 8% to 32% in numerous reports [2]. With the advent of MRI, the detection of acute herniated discs and ligament injuries, as well as intramedullary and extramedullary injuries in cases previously classified as SCIWORA, has increased [3,4]. In the majority of situations, SCIWORA is caused by hyperextension or hyperflexion injuries because of characteristic anatomic variations such as elasticity and a more deformable spine in children. They might initiate a transient occlusion of the vertebral arteries or the anterior spinal artery, resulting in a spinal infarction [3]. SCIWORA exhibits a wide range of neurological deficits, ranging from minor and transient injuries to complete spinal cord injuries. Neurological deficits can appear late, hours to days after injury [1]. We present the case of a patient who was diagnosed with a real SCIWORA along with a review of the literature.

## Case presentation

**Figure 1:**
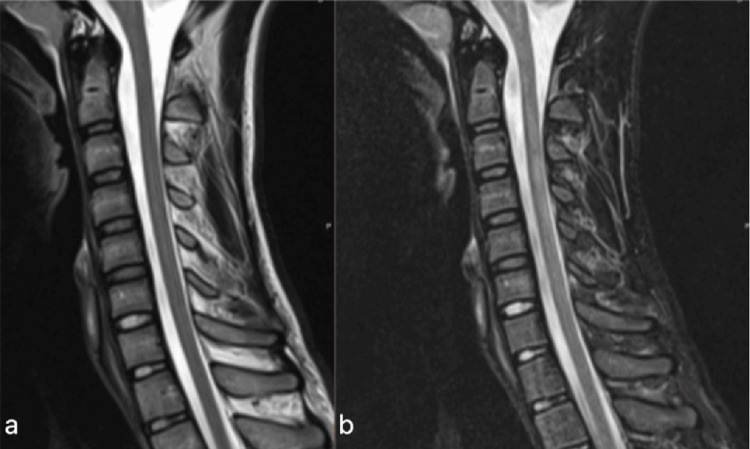
Cervical MRI scan showing no appreciable changes. a: T2 sagital. b: short tau inversion recovery sagittal

A 17-year-old male patient, a victim of a fall with traumatic brain injury and spinal trauma aftershock (heading duel) during a soccer game, presented to the emergency department of a tertiary hospital. He had occipital cranioencephalic trauma resulting from dorsal, cervical, and head impact after the fall. He denied loss of consciousness, nausea, or vomiting. A few minutes later, he reported a decrease in strength in the right upper limb and lower limbs, as well as changes in sensation in the right hemibody. He was admitted to the emergency room on a spine board stretcher with a Glasgow Coma Scale score of 15 and scored level D on the American Spinal Injury Association (ASIA) scale. He underwent a detailed neurological examination, which revealed right lower limb monoparesis, with a muscular strength grade of 3/5, without left limb deficits. No sensation or vesico-sphincter changes were reported. The remaining neurological examination was unremarkable. He underwent a CT of the head and spine, followed by an MRI of the dorsal/cervical spine. No abnormalities were found (Figure 1). Given the attained deficit, the patient was admitted to the pediatric neurosurgical ward. Based on the clinical history and imaging findings, real SCIWORA was diagnosed. He remained in the hospital for eight days, maintaining the same neurological status. During this period, electromyography was unremarkable. The patient was discharged from the hospital for elective admission to a rehabilitation center. Neuroaxis MRI examination 1.5 months later showed normal findings. At the reassessment consultation two months later, the patient exhibited complete reversal of symptoms, having returned to his normal life without limitations and with former sports activity.

## Discussion

Since the first description of SCIWORA by Pang and Wilberger in 1982, the definition of this entity has constantly evolved. This change is largely due to the evolution of diagnostic methods, namely, MRI, which began to detect changes that were not previously reported [5]. Although not yet universally accepted, the literature distinguishes three designations in pediatric patients (Table 1) [6].

**Table 1:** Types of SCIWORA. SCIWORA: spinal cord injury without radiological abnormality; MRI: magnetic resonance imaging

SCIWORA	Non-SCIWORA spinal cord injury	Real SCIWORA
Intrinsic cord signaling abnormalities can be seen on MRI (it only applies to children without congenital abnormalities of the spine)	Spinal cord injury without plain radiographic anomaly: this incorporates extrinsic injuries, for example, epidural hematomas, traumatic disk herniations, and ligament ruptures	Spinal cord injury without neuroimaging abnormality: it is used when the results of all modalities, including MRI, are negative; the prognosis is exceptional for these patients

SCIWORA represents a moderately infrequent disease; however, it may be a devastating injury among children. Piatt found that SCIWORA was responsible for nearly 20% of injuries in children under three years old. Between those aged 3-12 and 13-20, an incidence of 9.4% and 5%, respectively, was reported [7]. Prior reports have verified that SCIWORA in youngsters usually occurs within the higher cervical spine, whereas in adolescents it is more typically seen in the lower cervical and thoracic spine. The severity of the injury is also significantly influenced by the patient’s age, with children below eight years old often sustaining complete and severe neurological deficits [8].

In our case, the patient presented with an incomplete neurological deficit. At the time of admission, he was showing partial neurological recovery, suggesting a good prognosis.

Knox found that, overall, the most common cause of injury was sports-related. It accounted for 41% of injuries, followed by motor vehicle accidents at 26%, falls at 14%, assault at 4%, and being hit with a falling object at 3% [8].

There are four supposed mechanisms for SCIWORA, namely, longitudinal distraction, hyperflexion, hyperextension, or ischemic spinal cord injury [9]. It is believed that SCIWORA is seen less often in adults as a result of age-related changes in bone morphology and a reduction in ligamentous laxity [10]. Toddlers also have proportionately larger heads with underdeveloped neck muscles. As a result, infants and young children are particularly vulnerable to hyperflexion and hyperextension injuries.

Patients diagnosed with SCIWORA have a wide range of neurological deficits, ranging from mild and transitory symptoms such as paresthesia in the fingers to quadriplegia. In some patients, symptoms appear at the time of injury. However, it should be noted that neurological deficits may not manifest until a few minutes after the injury (they can develop very slowly over time as swelling or bleeding occurs around the injured level).

Initial clinical evaluation and history-taking are more difficult in young patients. Consequently, initial treatment begins with spinal immobilization and clinical assessment.

After the initial management in the field, the diagnostic workup of patients suspected of having spinal cord injury should begin with a detailed medical history, possibly obtained from eyewitnesses, to determine the mechanism of injury. Conventional radiographs are generally performed as a first-line imaging test to exclude fractures or subluxations. CT scans are more accurate in detecting vertebral fractures. MRI is the best modality for direct evaluation of the spinal cord [11,12]. We reviewed the literature and adapted the diagnostic protocol from Atesok et al. (2018) with minor changes (Figure 3). We emphasize the importance of including peripheral nerve pathology in the differential diagnosis. Follow-up MRI at six to nine months should also be considered.

**Figure 2:**
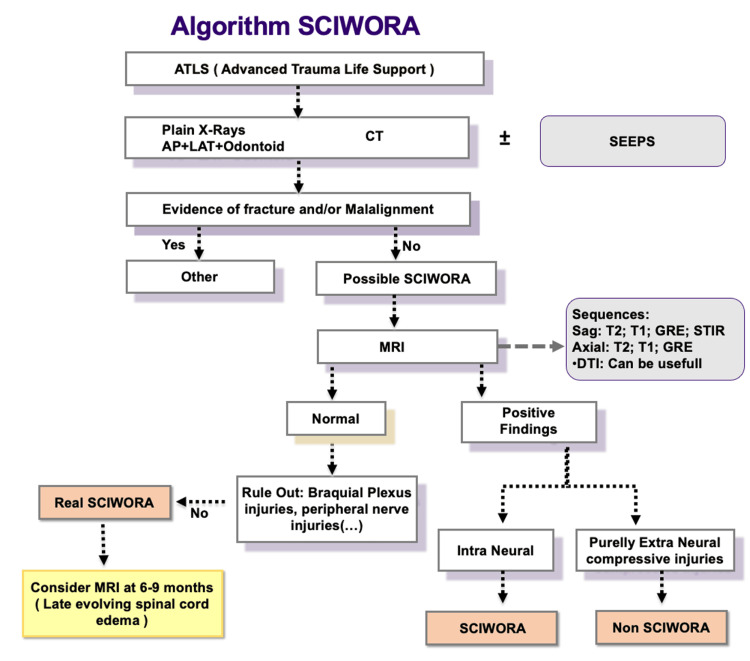
SCIWORA algorithm. Adapted from Atesok et al. [11]. SSEP: somatosensory evoked potentials; GRE: gradient echo; STIR: Short tau inversion recovery; AP: anteroposterior; LAT: lateral; CT: computed tomography; MRI: magnetic resonance imaging; SCIWORA: spinal cord injury without radiological abnormality

Spinal cord lesions detected by MRI are important prognostic factors in patients with SCIWORA. Small hematomas (up to one-third of the spinal cord diameter) or edema have a good prognosis and in most cases disappear with time. Anatomic transection of the spinal cord or considerable hematomas (more than half the diameter of the spinal cord) have a poor prognosis and clinically manifest as paresis or paralysis [12]. Patients with neurological findings consistent with SCIWORA on examination and a normal MRI generally have an excellent prognosis [10]. Another important factor in predicting prognosis is the neurological status. ASIA is the currently used tool for assessing neurological impairment in patients with SCIWORA. The literature shows that patients with less severe spinal cord injuries are more likely to achieve a full recovery. However, patients with severe injuries were found to have residual or persistent neurological dysfunction at follow-up [12,13].

Based on the current knowledge and past literature, surgical treatment is not recommended in SCIWORA patients with pure or normal intraneural MRI, regardless of neurological status. Clear MRI evidence of ligament injury, spinal cord compression, and instability, along with declining neurological findings, should be indications for surgical decompression with or without fusion [11]. There is a lack of data supporting the regular use of high-dose intravenous steroids in SCIWORA patients [10]. The main treatment option for patients with spinal cord injuries is external spinal immobilization for up to 12 weeks. Patients are also advised to avoid high-risk activities for six months to prevent the worsening of symptoms and reduce the risk of further injury [12]. Physiotherapy can be started as soon as the patient’s general condition allows and can continue throughout the treatment [10].

Discharging a patient with a motor disability can be a challenge for the family and the patient. In our case, rapid admission to a rehabilitation program and reassurance was fundamental. The post-traumatic stress disorder (PTSD) rate for spinal cord injuries is between 7% and 44%. In this case, the patient in question did not need psychobehavioral therapy, as the deficit had improved completely. In specific cases, we should consider offering reak SCIWORA patients a psychological co-treatment [3].

## Conclusions

In patients with SCIWORA, MRI is crucial and should be done for all patients. Imaging findings are extremely important in predicting outcomes. A better understanding of the disease progression can help the doctor to diagnose accurately and plan management with improved clarification and education for the frustrated patient and their families. This case report emphasizes the good prognosis for patients in whom MRI is normal. Psychological outcomes resulting from neurologic incapacity and trauma are tremendous and psychological care is advisable to prevent PTSD.
